# Ferroptosis and Charcot–Marie–Tooth Disease 1A: Emerging Evidence for a Pathogenic Association

**DOI:** 10.3390/antiox14030331

**Published:** 2025-03-11

**Authors:** Jacob B. White, Kayla L. Sanchez, Antonio Currais, David Soriano-Castell, Pamela Maher, Salvador Soriano

**Affiliations:** 1 Department of Pathology and Human Anatomy, School of Medicine, Loma Linda University, Loma Linda, CA 92354, USA; jwhite01@students.llu.edu (J.B.W.); klsanchez@students.llu.edu (K.L.S.); 2 The Salk Institute for Biological Studies, 10010 North Torrey Pines Road, La Jolla, CA 92037, USA; acurrais@salk.edu (A.C.); dsorianocastell@salk.edu (D.S.-C.)

**Keywords:** Charcot–Marie–Tooth, CMT1A, ferroptosis, peripheral neuropathy, oxidative stress, ferroptotic stress, lipid peroxidation, PNS, neurodegeneration

## Abstract

Charcot–Marie–Tooth disease (CMT) is the most common hereditary peripheral neuropathy worldwide, presenting clinically as muscle weakness that progresses to impaired ambulation or quadriplegia with age. CMT1A, the most common subtype, is caused by a duplication in PMP22, encoding an essential membrane protein for Schwann cell myelin integrity. While the mechanisms of peripheral neurodegeneration in CMT1A are poorly understood, excessive oxidative stress, particularly lipid peroxidation, is a known pathological feature, and antioxidant therapy has reversed the CMT1A phenotype in a mouse model. For the first time, we define the pathogenic link between CMT1A and ferroptosis, a form of regulated cell death caused by excessive lipid peroxidation and hindered antioxidant defenses. Human-derived CMT1A fibroblasts showed greater susceptibility to RSL3, a pro-ferroptosis agent, compared with controls, alongside several ferroptosis markers, including elevated lipid peroxides and depleted GPX4, a critical anti-ferroptosis repressor. Similarly, transcriptomic analysis of human iPSC-derived Schwann cells revealed elevated ferroptosis activation and cellular stress markers in CMT1A. We propose that chronic, sublethal ferroptotic stress, mediated by lipid peroxide accumulation, depletes antioxidant defenses in CMT1A Schwann cells, leading to decompensation with age, manifesting as symptomatic disease. These results emphasize ferroptosis as a driver of CMT1A pathology, potentially revealing a new therapeutic path.

## 1. Introduction

Charcot–Marie–Tooth disease (CMT) is the most common hereditary peripheral polyneuropathy, with a prevalence of 126,000 individuals in the United States and 2.6 million people worldwide [1]. CMT, a genetically and clinically heterogeneous condition, is caused by over 100 mutations, most of which lead to progressive motor weakness with the relative sparing of respiratory muscles [2]. This condition often results in quadriplegia by late adulthood [2]. Accounting for >50% of CMT cases is type 1A, caused by a 1.4 Mb duplication in the PMP22 gene on chromosome 17, encoding the glycoprotein peripheral myelin protein 22 [3]. While the precise function of the PMP22 protein is currently unknown, recent publications indicate its vital role in myelin sheath homeostasis and anterograde cholesterol trafficking within Schwann cells [4]. Histological examination of peripheral nerves in CMT1A patients shows onion bulb formations, which are concentric layers of collagen and Schwann cell processes caused by repeated cycles of demyelination and remyelination [5]. The molecular processes underlying Schwann cell dysfunction or death in CMT1A remain poorly understood [6]. 

Excess oxidative stress in neural tissue has emerged as a key pathogenic mechanism in several neurological diseases, with CMT serving as a prominent example [7]. Increased levels of serum oxidative stress markers, including lipid-based reactive oxygen species (ROS), have been reported in CMT1A patients [7]. Moreover, the administration of the antioxidant curcumin to transgenic CMT1A mice improved the symptoms of muscle weakness [8]. Cultured Schwann cells from these transgenic CMT1A mice also showed increased total ROS, increased mitochondrial ROS, and decreased mitochondrial membrane potential compared with cells from wild-type mice [8]. Furthermore, a severe early-onset form of CMT, type 4A, showed comparable symptoms to late-stage CMT1A and further highlights the role of oxidative stress in disease pathogenesis [9]. CMT4A is caused by mutations in the GDAP1 gene, which encodes a protein in the outer mitochondrial membrane (OMM) with domains analogous to glutathione-S-transferases (GSTs) [9,10]. Mechanistic studies have indicated that CMT4A patient-derived fibroblasts show reduced levels of GDAP1, correlating with decreased intracellular glutathione and mitochondrial membrane potential [9,10]. Curiously, the upregulation of wild-type GDAP1 in neuronal HT22 cells protects against the glutathione depletion caused by oxidative glutamate toxicity [10]. 

Over the past two decades, studies on non-excitotoxic receptor-mediated glutamate toxicity have revealed an oxidative, regulated cell death which is now referred to as the ferroptosis pathway [11,12]. In ferroptosis, the depletion of cellular antioxidant defenses, especially glutathione, leads to a critical accumulation of lipid peroxides (LPOs), which activates cellular stress responses and can potentially result in cell death [11,12,13,14,15,16]. The most extensively characterized mechanisms of ferroptosis induction involve the inhibition of either the cystine/glutamate antiporter (system Xc-; xCT), which imports the precursor metabolite for glutathione synthesis, or glutathione peroxidase 4 (GPX4), an enzyme that catalyzes the reduction of lipid peroxides using reduced glutathione [11,12,13,14,15,16]. Although often compared to forms of cell death, such as apoptosis and necrosis, the ferroptosis pathway shows a degree of reversibility [17]. While cells undergoing sublethal ferroptotic stress exhibit no apparent gross morphological changes, those undergoing ferroptotic cell death display cellular swelling, mitochondrial shrinkage with reduced cristae, and plasma membrane rupture [11,18]. 

Ferroptosis and its pertinent hallmarks, including reductions in glutathione levels and elevated LPO, have been implicated in the pathogenesis of several neurological diseases, including Alzheimer’s dementia, Parkinson’s disease, traumatic brain injury, and stroke [13,19,20,21]. Recent evidence implicates sublethal, chronic ferroptotic stress in the activation of microglia, which themselves contribute to the progression of neurological diseases, including AD, PD, and amyotrophic lateral sclerosis (ALS) [14,15,16,22,23,24]. Furthermore, the anti-ferroptotic compounds J147 and CMS121 have shown promising results in reversing cognitive decline in Alzheimer’s disease mice and have undergone phase 1 clinical trials for the treatment of Alzheimer’s disease patients (NCT03838185 and NCT05318040) [25,26,27,28,29,30,31]. 

**Figure 1:**
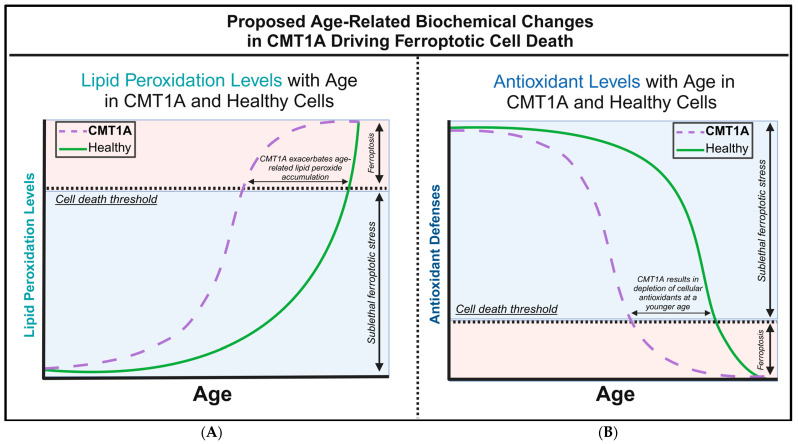
Our proposed hypothesis of the biochemical alterations driving ferroptotic cell death during aging in Charcot–Marie–Tooth disease type 1A (CMT1A) and healthy cells. (**A**) The accumulation of lipid peroxides increases with age as antioxidant homeostatic mechanisms gradually fail, leading to an exponential rise in lipid peroxidation during old age in healthy cells. In CMT1A, compromised antioxidant defenses result in a higher baseline rate of lipid peroxidation, causing CMT1A cells to reach the exponential decompensation point earlier than in healthy cells. (**B**) Intracellular antioxidant components, particularly glutathione and GPX4, effectively regulate lipid peroxidation until cellular defenses begin to decompensate in old age. In CMT1A, a PMP22 duplication leads to lower antioxidant levels from a young age, accelerating the decompensation against lipid peroxidation, which results in earlier cell death and symptom onset compared to healthy individuals.

There are currently no studies examining ferroptosis in the hereditary diseases of the peripheral nervous system (PNS), including any forms of CMT. However, although not previously identified as such, CMT1A patients exhibit several biochemical markers suggestive of ferroptosis pathway activation, including increased baseline lipid peroxides and decreased glutathione in serum, both of which were normalized with antioxidant therapy [7]. In addition, the treatment of transgenic CMT1A mice with curcumin, the chemical precursor of the anti-ferroptotic compound J147, significantly improved the symptoms of weakness [8,25]. Building on these studies, we hypothesized that chronic ferroptotic stress, in the form of lipid peroxide accumulation, accelerates cell death in CMT1A through the decompensation of antioxidant systems (Figure 1). To test this hypothesis, we exposed cultured primary fibroblasts from CMT1A and wild-type (WT) patients to RAS-selective lethal 3 (RSL3), a potent and specific inducer of ferroptosis that inhibits GPX4, and assessed the differences in cell death progression between the groups [32,33,34]. Levels of GPX4 and lipid peroxidation were also measured at baseline and following the induction of ferroptosis with RSL3 [32,33,34]. Additionally, we conducted in silico analysis using publicly available transcriptomic data from iPSC-derived Schwann cells of CMT1A and WT patients. This large-scale bioinformatics approach complemented our in vitro findings by identifying dysregulated ferroptosis-related genes and pathways in CMT1A. To our knowledge, these findings provide the first evidence linking ferroptosis to the pathogenesis of CMT, thereby identifying this pathway as a potential therapeutic target for CMT treatment.

## 2. Materials and Methods

### 2.1. Cell Culture 

Human-derived fibroblast cultures were purchased from the Coriell Institute for Medical Research (Camden, NJ, USA) and included two CMT1A cell lines (40-year-old GM05146 and 51-year-old GM05165) and two age-matched wild-type cell lines (40-year-old GM25932 and 51-year-old AG13230). Previous mechanistic studies using these CMT1A fibroblasts confirmed the presence of the PMP22 gene duplication (17p) using multiplex ligation-dependent probe amplification [4,35]. All of the cell lines were grown in Dulbecco’s Modified Eagle Medium (DMEM) with 10% heat-inactivated fetal bovine serum (FBS) and 10 µg/mL penicillin/streptomycin in a cell culture incubator (37 °C, 5% CO_2_, and 95% humidity). All cell culture reagents were purchased from Thermo Fisher Scientific (Waltham, MA, USA). Cell cultures grew to 80–100% confluency before splitting for the experiments. 

### 2.2. Phase-Contrast Microscopy and Cell Viability Counts 

Primary fibroblasts from CMT1A and healthy WT patients were seeded on 6-well cell culture plates at a density of 30,000–40,000 cells/well and placed in a cell culture incubator for 12–72 h. Cells were then treated with 100 nM of RSL3 (HY-100218A, MedChemExpress, Monmouth Junction, NJ, USA) for 4, 8, and 24 h [32,33,34]. Images of the cell cultures were taken at 4× and 10× magnifications. All of the images were captured using an Invitrogen™ EVOS™ FL Digital Inverted Fluorescence Microscope (Fisher Scientific, Waltham, MA, USA). For cell viability counting, the fibroblasts were considered to be undergoing ferroptotic cell death if they showed a rounded morphology and either increased birefringence or fully retracted cellular appendages. To distinguish from mitotic figures, each group was normalized to its corresponding untreated control wells. For the 51- and 40-year-old human CMT1A and WT primary fibroblasts, data were derived from *n* = 3 and *n* = 1 independent experiments with triplicates, respectively, which were supplemented with an MTT cell viability assay for all time groups. MTT viability assays were performed with a working concentration of 0.5 mg/mL MTT at 3 h, and formazan crystals were dissolved with 5% SDS in DMSO. 

### 2.3. Cell Lysis and Protein Isolation 

To perform cell lysis and protein isolation, CMT1A and control fibroblasts were plated on 6-well culture plates at a density of 30,000–40,000 cells/well and placed in a cell culture incubator for 12–72 h. Cells were then treated with either medium alone (vehicle control) or 100 nM of RSL3 for 8 h. After aspirating the treatment media, the cells were washed once with ice-cold phosphate-buffered saline (PBS) before adding 100 μL of radioimmunoprecipitation assay buffer (RIPA) with protease inhibitor cocktail added (Thermo Fisher Scientific, Waltham, MA, USA). Adherent cells were agitated for 30 min at 4 °C and scraped with a rubber policeman. Cell lysis samples were sonicated thrice for 2 s each, then centrifuged at 15,000 rpm for 30 min. Supernatant was isolated and frozen at −20 °C for future analysis. 

### 2.4. Western Blotting and Antibodies 

To perform Western blots, stored CMT1A and WT cell lysates were first separated by SDS-PAGE using Bolt™ Bis-Tris Plus Mini Protein Gels, 4–12% (Thermo Fisher Scientific, Waltham, MA, USA). Separated proteins were transferred from the SDS-PAGE gel to a nitrocellulose membrane and blocked with 5% nonfat powdered milk for 1 h. Membranes were washed with tris-buffered saline with 0.1% Tween^®^ 20 (TBST) and incubated overnight with primary antibody (1:1000 dilution) at 4 °C. After 24 h, the membranes were washed with TBST 3× for 5 min each, and then incubated with secondary antibody (1:10,000 dilution) for 1 h at room temperature. For the 51-year-old human primary fibroblasts cell lines, data were from *n* = 3 independent cell lysis experiments in triplicate. Membrane imaging was performed with an LI-COR Odyssey CLx and quantified using Image Studio 6.0 software. Raw membrane images are provided in Supplementary Materials (Supplementary Figure S7). 

All western blots used PageRuler™ Plus Prestained Protein Ladder (Thermo Fisher Scientific, Waltham, MA, USA) for protein size standards. Anti-β-actin antibodies (#ab8226, Abcam, Waltham, MA, USA; Thermo Fisher Scientific, Cat# MA1-140, Waltham, MA, USA) were used for the protein detection and normalization. Antibodies against GPX4 (#ab125066, Abcam, #, Waltham, MA, USA) were also used for protein detection. Secondary antibodies were used to visualize primary antibodies, and were purchased from Fisher Scientific (Waltham, MA, USA), and included 0.5 mg of donkey anti-mouse 680 nm (Cat# NC0250903), 0.5 mg of donkey anti-mouse 800 nm (Cat# NC9744100), 0.5 mg of donkey anti-rabbit 800 nm (Cat# NC9523609), and 0.5 mg of donkey anti-rabbit 680 nm (Cat# NC0250902).

### 2.5. Lipid Peroxidation Staining and Quantitation 

Human patient CMT1A and WT primary fibroblasts were seeded on 6-well culture plates at a density of 40,000 cells per well and placed in a cell culture incubator for 24–48 h. Fibroblasts were treated with either growth medium (vehicle control) or 100 nM of RSL3 for 8 h. After 8 h, the control or treatment medium was aspirated and replaced with control medium with 1 µM of BODIPY™ 581/591 C11 undecanoic acid. Plates were incubated for 30 min, and then washed 3× with PBS and fixed with 4% paraformaldehyde. Data from the 51- and 40-year-old cell lines were derived from *n* = 2 and *n* = 1 independent experiments, respectively, with triplicates for each treatment group. Both green and red emission values were collected using ImageJ software version 1.53t (https://imagej.net/ij/), and green/red ratios were calculated in Microsoft Excel version 2501 [36]. 

### 2.6. Statistical Analysis 

The Shapiro–Wilk test, Mann–Whitney U test, and Grubbs’ test were performed using online calculators [37,38,39]. All other statistical analyses, including unpaired Student’s *t*-test, F-test, and calculations of standard error, were performed using Microsoft Excel software version 2501. A *p*-value of < 0.05 was considered statistically significant for all statistical tests. For Grubbs’ test, statistical significance for identifying outliers was defined as a *p* < 0.05 or a Z-score > 2, and pertinent data points were excluded from further analysis. 

### 2.7. Transcriptomic Analysis 

All transcriptomic data were retrieved from the NCBI’s Gene Expression Omnibus (GEO) [40]. The first transcriptomic set included only hiPSC-derived Schwann cells, excluding human embryonic cell lines (hESC); all other replicates were included in the analyses (GEO Accession: GSE69988) [41]. The second transcriptomic dataset analyzed only mature differentiated Schwann cells, also excluding less-differentiated stem cells (GEO Accession: GSE97851) [42]. For the Gene Set Enrichment Analysis (GSEA), the following analysis parameters were used: enrichment statistic—weighted; ranking metric—log2_Ratio_of_Classes; gene list sorting mode—real; gene list ordering mode—descending [43]. All gene sets used in the GSEA were retrieved from the FerrDb V2 website [44]. For the DEG analysis, the aforementioned datasets were analyzed with GEO2R with the following parameters: *p*-value adjustment—Benjamini and Hochberg; log transformation—auto-detect; apply limma precision weights (vooma)—no; force normalization—no. Afterwards, the DEGs were ranked by the adjusted *p*-value, and selected for the same genes used in the GSEA. A custom R script was used to rank the DEGs by ascending adjusted *p*-values, whilst also showing fold changes, and graphs were constructed with the R module ggplot2 [45]. For QIAGEN’s Ingenuity Pathway Analysis (IPA), only the first transcriptomic dataset was used, with the cutoff for the adjusted *p*-value set at <0.05 and the log fold change set to <−0.5 and >0.5 [46]. A custom pathway using the significant DEGs analyzed with GEO2R was constructed in IPA, with the previous analysis overlayed on the pathway.

## 3. Results

### 3.1. CMT1A Fibroblasts Show Greater Vulnerability to RSL3-Induced Ferroptosis Compared with Age-Matched Controls

**Figure 2:**
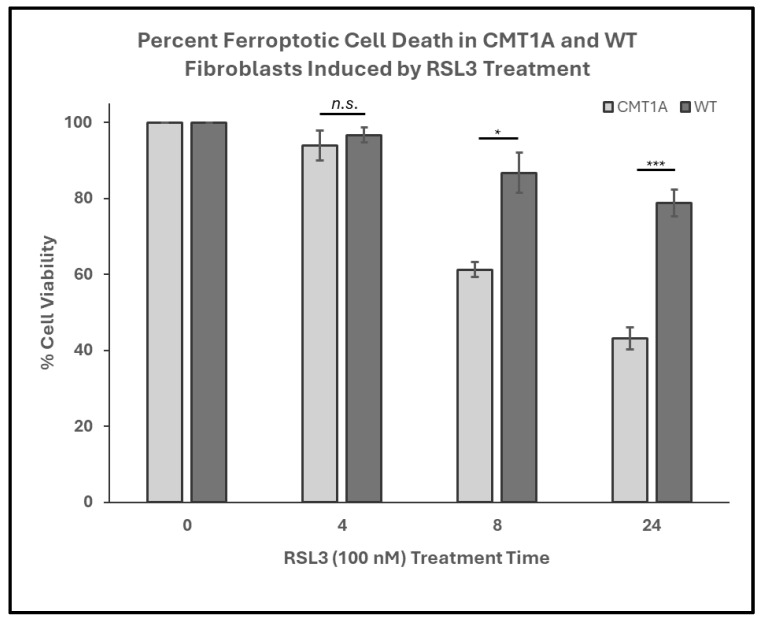
Percent cell viability of Charcot–Marie–Tooth disease type 1A (CMT1A) and wild-type (WT; control) primary fibroblasts derived from 51-year-old human patient donors at 0-24 h of treatment with RAS-selective lethal 3 (RSL3), a ferroptosis inducer. Cells were seeded at 40,000 cells/well for 24–72 h. Cell medium was then aspirated and replaced with 100 nM of RSL3. After 4, 8, and 24 h, the cells were imaged using an Invitrogen EVOS Digital Color Fluorescence Microscope at 4× and 10× magnifications. Data were derived from *n* = 3 independent experiments. Cell counts were performed manually for all groups, and statistical analysis was performed in Microsoft Excel. Error bars signify the standard error of the mean. * *p* < 0.05, and *** *p* < 0.001; n.s. = no significance.

In our working model of CMT1A pathology, we hypothesize that affected cells experience higher levels of basal ferroptotic stress, compromising their ability to defend against both exogenous and endogenous sources of ROS (Figure 1). We reason that, if higher levels of lipid peroxidation are present in CMT1A cells, this places them closer to the ferroptosis cell death threshold, as depicted in Figure 1. The additional ferroptotic stress generated by RSL3 would lead to more CMT1A cells crossing that threshold compared to age-matched controls. To test this hypothesis, we assessed the susceptibility of cultured human CMT1A fibroblasts to pro-ferroptotic compounds in comparison with age-matched controls (WT). Specifically, we treated primary dermal fibroblasts from CMT1A patients and aged-matched WT controls with RSL3 [32,33,34]. Initial MTT cell viability assays at 4, 8, and 24 h of RSL3 treatment revealed 100 nM as the lowest dose that reached a significance threshold of *p* < 0.001 between CMT1A and WT at 8 h, reflecting highly differential cell death (Supplementary Figure S1). This dose has been used frequently in other studies, and thus was used as the effective dose in subsequent experiments [47,48]. To evaluate differences in the progression of ferroptotic cell death, primary human fibroblasts from 51- and 40-year-old CMT1A patients and age-matched WT controls were exposed to 100 nM of RSL3 for intervals of 4, 8, and 24 h, and living or dead cells were counted manually after the images were captured. At 8 and 24 h, the CMT1A fibroblasts exhibited a significantly higher percentage of cytotoxicity as compared with the WT fibroblasts (Figure 2). At 0–4 h, no significant differences were observed between the CMT1A and WT cells (Figure 2). These results were supported by an MTT biochemical assay (Supplementary Figure S2), and cell counting data were verified with the 40-year-old cell lines (Supplementary Figure S3), both showing comparable results. These data indicate a greater susceptibility to pro-ferroptotic stress in human CMT1A fibroblasts over WT cells, with susceptibility differences appearing after 8 h of treatment (Figure 2). Within our working model, these findings support the concept that elevated lipid peroxidation and/or reduced antioxidant levels in CMT1A cells contribute to premature cell death (Figure 1).

**Figure 3:**
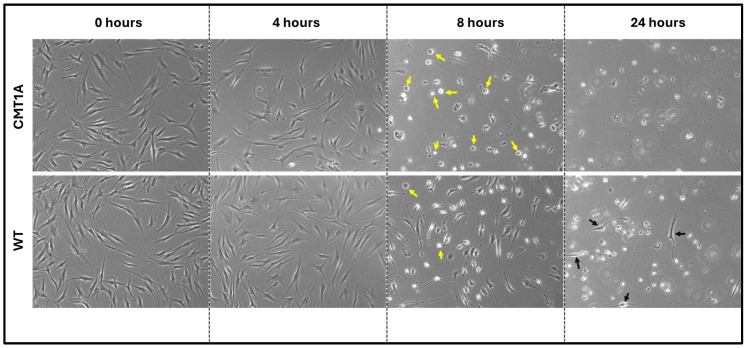
Phase-contrast microscopy images (4× magnification) of 40-year-old CMT1A and wild-type (WT; control) fibroblasts derived from human subjects. Cells were seeded at 40,000 cells/well for 24–72 h. Cell medium was then aspirated and replaced with 100 nM of RSL3. After 4, 8, and 24 h, the cells were imaged using an Invitrogen^TM^ EVOS^TM^ FL Digital Inverted Fluorescence Microscope at 4× magnification. Yellow arrows denote the ferroptotic morphology, while black arrows indicate living cells.

Fibroblasts undergoing ferroptosis show gross morphological characteristics such as membrane retraction and the loss of surface attachment [11,17]. Visual representation of these morphological changes is shown in Figure 3. Changes are apparent at 8 h of the 100 nM RSL3 treatment, and, after 24 h, both CMT1A and WT fibroblasts appeared as small, unattached spheres distinct from the living spindle-shaped fibroblasts (Figure 3). At 24 h, no CMT1A cells were adherent, while some WT cells remained (Figure 3, black arrows). These results are also consistent with our hypothesis, in that an inciting pro-ferroptotic stressor, RSL3, causes greater cell death at comparable doses in CMT1A cells than in WT cells, suggesting an underlying compromise of anti-ferroptotic defenses at the same age (Figure 1A).

### 3.2. Changes to GPX4 and Lipid Peroxidation Emphasize Ferroptosis Vulnerability in CMT1A

**Figure 4:**
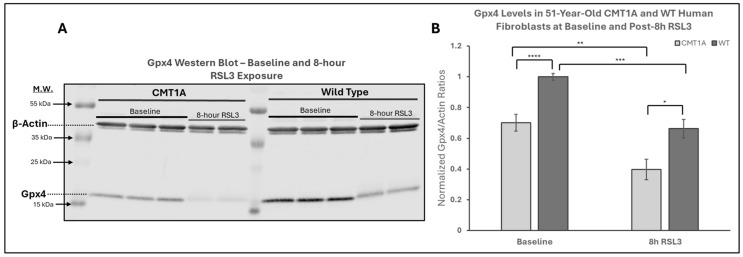
Western blot analysis of glutathione peroxidase 4 (GPX4) in human-derived fibroblasts from a 51-year-old Charcot–Marie–Tooth disease type 1A (CMT1A) patient and a wild-type (WT) control subject. GPX4 is a key inhibitor of the ferroptosis pathway, using reduced glutathione (GSH) to neutralize lipid peroxides (LPOs). RSL3 inhibits GPX4, promoting ferroptosis [32]. (**A**) Representative Western blot images of GPX4 and actin (loading control) in cells treated with either medium alone (vehicle control) or 100 nM of RSL3 for 8 h. Cell lysates were separated by SDS-PAGE and transferred onto a nitrocellulose membrane, followed by incubation with primary antibodies (1:1000, 24 h, 4 °C) and secondary antibodies (1:10,000, 1 h, room temperature). (**B**) Densitometric quantification of the GPX4 band intensity, normalized to β-actin, from Western blot membranes using Image Studio 6.0. Data were from *n* = 3 independent cell lysis experiments in triplicate or duplicate. Error bars denote the standard error of the mean. Treatment group averages were normalized to the WT baseline average. Student’s unpaired *t*-test or the Mann–Whitney U test was used to calculate statistical differences. * *p* < 0.05, ** *p* < 0.01, *** *p* < 0.001, and **** *p* < 0.0001.

The above data are consistent with our hypothesis that there is a higher level of pro-ferroptotic stress and/or a deficiency in antioxidant defenses in CMT1A cells that render them more vulnerable to advancing into cell death through ferroptosis (Figure 1). If that hypothesis is correct, CMT1A cells could be expected to display lower levels of GPX4 and, consequently, higher levels of LPO. GPX4 acts as a critical repressor of ferroptosis in its ability to use GSH to detoxify LPO, and its inhibition directly causes the progression to cell death through ferroptosis [11,12,18,32]. To examine this anti-ferroptotic mechanism, we performed Western blotting of CMT1A and WT fibroblasts after 8 h of the 100 nM RSL3 treatment (Figure 4). This revealed significantly reduced GPX4 expression in CMT1A over WT at baseline (*p* < 0.0001) and after 8 h of RSL3 treatment (*p* = 0.019). Within each of the CMT1A and WT groups, the GPX4 levels decreased significantly after 8 h of RSL3 treatment from their baseline (*p* = 0.0022 and *p* = 0.00011, respectively). Overall, these results suggest that CMT1A fibroblasts may have weakened anti-ferroptotic defenses due, at least in part, to reduced GPX4 levels, and this vulnerability is further exacerbated when cells are exposed to RSL3, likely reflecting the visual cell death seen at 8 h (Figure 3).

**Figure 5:**
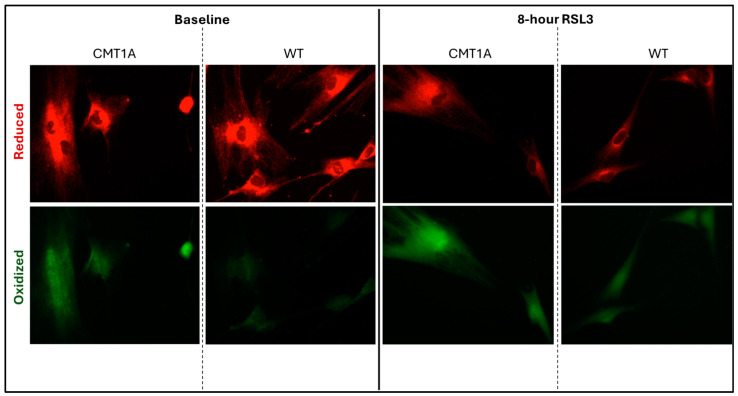
Fluorescent microscopy images (10× magnification) of 51-year-old Charcot–Marie–Tooth disease type 1A (CMT1A) and wild-type (WT; control) human-derived fibroblasts stained with BODIPY™ 581/591 C11 undecanoic acid, a lipid peroxidation sensor. BODIPY-C11 acts as a lipid peroxidation sensor, as the oxidation of this lipid’s polyunsaturated butadienyl moiety shifts the fluorescence emission peak from red (590 nm) to green (510 nm). After the CMT1A or WT cells were treated with medium alone (vehicle control) or 100 nM of RSL3 for 8 h, BODIPY-C11 staining was performed at 37 °C in a cell incubator for 30 min before fixation with 4% paraformaldehyde.

**Figure 6:**
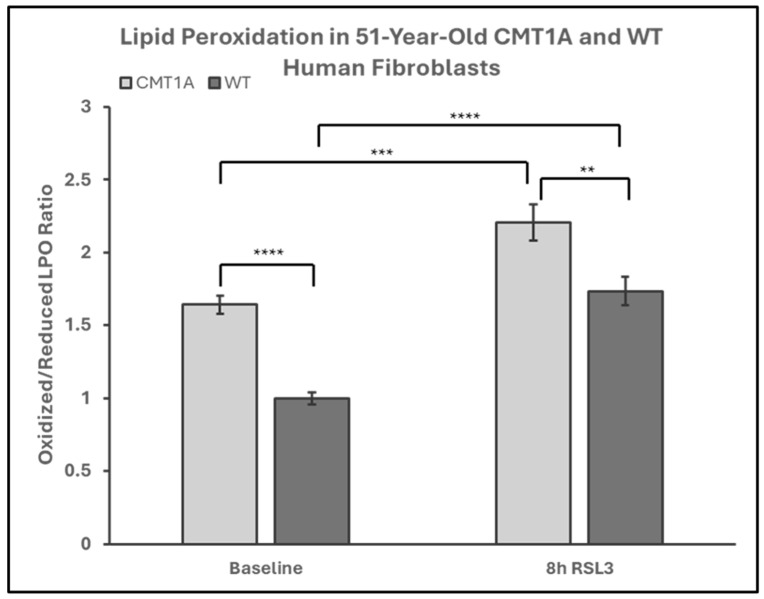
Quantification of the green-to-red emission (oxidized-to-reduced) ratio from BODIPY™ 581/591 C11 undecanoic acid staining of 51-year-old Charcot–Marie–Tooth disease type 1A (CMT1A) and wild-type (WT; control) human-derived fibroblasts, before and after ferroptosis induction with RSL3. Fibroblasts were treated with either growth medium (vehicle control) or 100 nM of RSL3 for 8 h, prior to a 30-min incubation at 37 °C with 1 μM of BODIPY™ 581/591 C11 and immediate fixation with 4% paraformaldehyde. Green and red emission images were captured with a digital fluorescent microscope and quantified in ImageJ software version 1.53t. Statistical testing was performed in Microsoft Excel using Student’s *t*-test or the Mann–Whitney U test. Data are from *n* = 2 independent experiments in triplicate. Treatment group averages were normalized to the WT baseline average. Error bars denote the standard error of the mean. ** *p* < 0.01, *** *p* < 0.001, and **** *p* < 0.0001.

Lower levels of GPX4 in a pro-ferroptotic environment would be expected to correlate with the accumulation of LPOs, which could ultimately lead to cell death. Therefore, we measured the LPO levels in CMT1A and WT patient-derived fibroblasts using BODIPY™ 581/591 C11 undecanoic acid (BODIPY-C11), a sensitive marker of lipid peroxidation, at baseline and after 8 h of the 100 nM RSL3 treatment. BODIPY-C11 staining revealed a markedly increased ratio of peroxidized lipids (green) to reduced lipids (red) in CMT1A cells as compared to WT cells (Figure 5 and Figure 6). This significant difference was maintained after 8 h of RSL3 treatment, although with marked increases in lipid peroxidation levels in both groups (Figure 5 and Figure 6). Comparable results were observed in the 40-year-old fibroblasts (Supplementary Figures S4 and S5). These data, showing substantial baseline differences between the CMT1A and WT cells of lipid peroxidation, further support our working hypothesis by directly showing increased lipid peroxidation in CMT1A cells at the same age as the non-diseased phenotype (Figure 1B). 

### 3.3. CMT1A Schwann Cells Significantly Overexpress Ferroptosis Pathway Genes

Our in vitro data indicate that fibroblasts from CMT1A patients are more susceptible to ferroptosis induction than WT cells (Figure 2, Figure 3, Figure 4, Figure 5 and Figure 6). Our interpretation of these findings is that CMT1A cells experience elevated ferroptotic stress, predisposing them to cell death at an earlier age than their non-diseased counterparts (Figure 1). Although fibroblasts, due to the broad expression of PMP22 in the human body, have been widely used as a model for mechanistic CMT1A research, they are not the primary cell type affected by the disease, and do not provide a suitable context to study neurodegeneration mechanisms in the PNS [4,35,49,50]. In contrast, Schwann cells overexpress PMP22 at markedly higher levels and are the primary cells to experience phenotypic effects in CMT1A [1,2,3,4,5]. To better model CMT1A disease and further explore the potential role of ferroptosis in its pathogenesis, we conducted a whole-pathway transcriptomic analysis. This approach enabled us to examine the expression trends of hundreds of ferroptosis-related genes, identifying patterns of overexpression or underexpression. We retrieved two transcriptomic datasets of iPSC-derived Schwann cells from CMT1A patients and WT controls using publicly available data from the NCBI’s Gene Expression Omnibus [40,41,42]. The first transcriptomic dataset, generated by Mukherjee-Clavin et al., was derived from Schwann cells from human induced pluripotent stem cells (hiPSCs) in two CMT1A patients (age 17 and 51) and one WT (control, age 61) patient [41]. The second transcriptomic dataset, generated by Shi et al., was derived from iPSC-generated Schwann cells from a symptomatic CMT1A patient and a control patient, both of an unspecified age [41]. The analysis results of Mukherjee-Clavin et al. are shown in the figures below, while the GSEA analysis of Shi et al. is shown in Supplementary Figure S6 [41,42]. 

**Figure 7:**
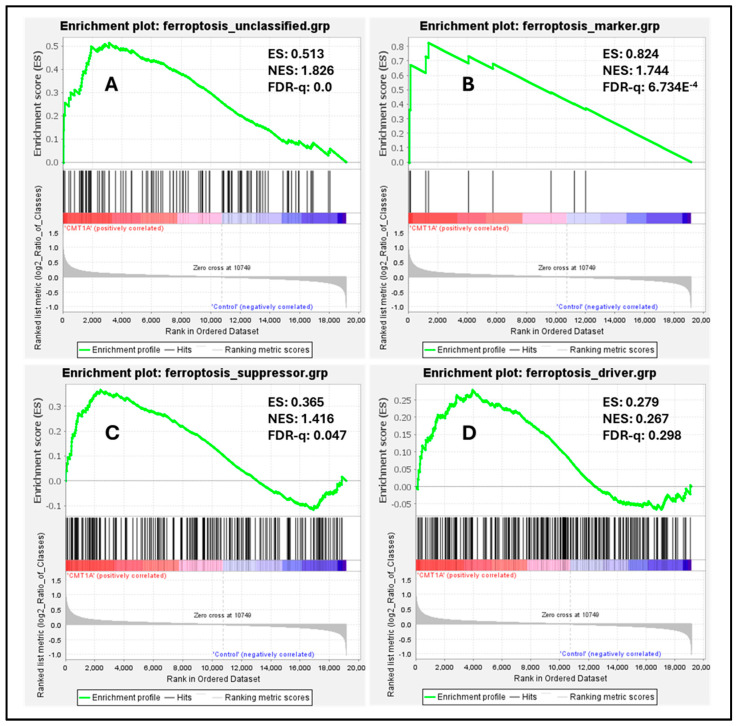
Enrichment plots of ferroptosis markers, suppressors, drivers, and unclassified genes in CMT1A iPSC-derived Schwann cells compared with wild-type (WT) control Schwann cells. Black colored marks indicate gene identifiers, while red and blue colors denote upregulated and downregulated gene transcripts, respectively, compared to controls. Transcriptomic data were retrieved from the NCBI’s Gene Expression Omnibus (GEO) website [40]. Data were generated by Mukherjee-Clavin et al. (2019) [41]. Based on the GSEA developer recommendation of a false discovery rate (FDR)-q < 0.25, unclassified ferroptosis regulators (**A**), ferroptosis markers (**B**), and ferroptosis suppressors (**C**) showed significant upregulation and enrichment compared with WT samples, but not ferroptosis drivers (**D**). Ferroptosis gene sets were retrieved from FerrDb V2.0 [44].

To measure the baseline activation of the ferroptosis pathway in human Schwann cells, we performed a Gene Set Enrichment Analysis (GSEA) on these datasets, a powerful method to identify significantly enriched gene sets based on upregulation or downregulation patterns, as quantified by the false discovery rate (FDR-q) value [43]. According to the GSEA developers, an FDR-q value of <0.25 denotes significant enrichment [43]. We focused our analysis on four distinct gene sets from FerrDb V2.0, the most comprehensive ferroptosis gene database currently available [44]. FerrDb V2.0 includes ferroptosis markers, which indicate the presence or absence of ferroptosis; ferroptosis drivers, which are genes promoting ferroptosis; ferroptosis suppressors, denoting genes blocking ferroptosis progression; and unclassified ferroptosis genes, which play an unclear role in ferroptosis [44]. In our analysis of the dataset by Mukherjee-Clavin et al., significantly upregulated genes included markers of ferroptosis presence (Figure 7B, FDR-q = 6.734 × 10^−4^), unclassified genes (Figure 7A, FDR-q = 0), and ferroptosis suppressors (Figure 7C, FDR-q = 0.047), with ferroptosis drivers not showing significant differences (Figure 7D, FDR-q = 0.298) [41,44]. Enrichment analysis of the separate dataset by Shi et al. showed comparable results, with significant upregulation of ferroptosis markers and suppressors, although neither unclassified ferroptosis genes nor ferroptosis drivers were significantly enriched in this dataset (Supplementary Figure S6) [42,44]. The significant upregulation of ferroptosis markers indicates the activation of the ferroptosis pathway in CMT1A Schwann cells, which is consistent with FerrDb V2.0’s description of ferroptosis markers identifying the presence of this pathway [44]. The upregulation of ferroptosis suppressors, alongside the presence of ferroptosis markers, likely reflects a compensatory response at the cellular level to sublethal ferroptotic stress, as described in Figure 1, and supported by our GPX4 (Figure 4) and LPO data (Figure 5 and Figure 6). There is a non-significant trend toward the enrichment of ferroptotic driver genes in CMT1A Schwann cells (Figure 7B, FDR-q = 0.298). Our interpretation of this result is that the impact of PMP22 duplication on antioxidant defenses, including the loss of GPX4, may trigger a negative feedback attempt to downregulate genes that would exacerbate further the progression to ferroptotic cell death. This dynamic balance suggests that, while CMT1A Schwann cells engage in protective compensatory mechanisms to counteract ferroptosis, this protection may weaken with age, which is also consistent with the sublethal stress model provided in Figure 1.

### 3.4. CMT1A Schwann Cells Differentially Express Genes Pertaining to Ferroptotic Stress

**Figure 8:**
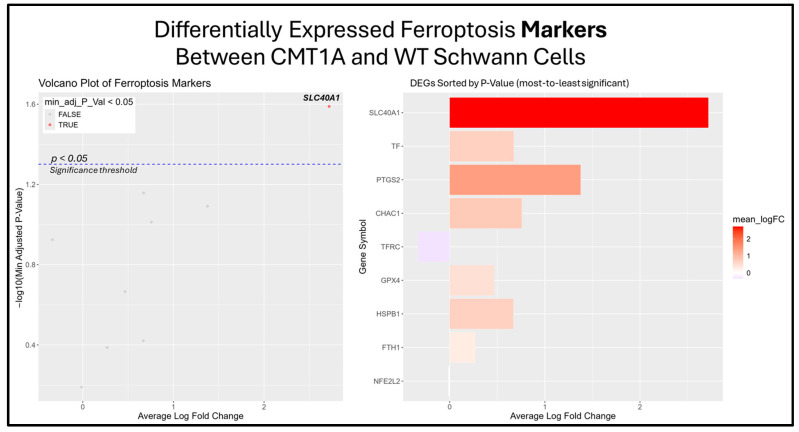
Volcano plots and bar graphs showing all of the significant differentially expressed genes (DEGs) between CMT1A and WT Schwann cells in the “Ferroptosis Markers” gene set, as listed on FerrDb 2.0 [44]. Transcriptomic data were obtained from Mukherjee-Clavin et al. (2019) [41]. DEGs with adjusted *p*-values and log fold changes (logFCs) were retrieved using GEO2R. The ggplot2 R module was used to graph the results [45]. For all analyses, significance was denoted as *p* < 0.05, represented by the dotted blue line in the volcano plot. In both graphs, the X-axis denotes positive (rightward) and negative (leftward) fold changes, while the Y-axis represents -log10(adjusted *p*-value), where higher values indicate greater significance. Labeled genes highlight the key ferroptosis suppressors differentially expressed in CMT1A Schwann cells.

The previous results suggest that the ferroptosis pathway exhibits increased activity in CMT1A Schwann cells due to the presence of elevated ferroptosis markers (Figure 7B). While the GSEA provides insight into whole-pathway enrichment, it does not directly test individual genes for statistical significance. To address this, we performed a differentially expressed gene (DEG) analysis to identify the primary genes responsible for the observed ferroptosis activation. After obtaining DEGs from the Mukherjee-Clavin et al. dataset with GEO2R, we sorted these genes into the following three significantly enriched datasets from FerrDb V2.0: ferroptosis markers, suppressors, and unclassified genes (Figure 7A–C) [40,41,44]. When ranked by significance (smallest to highest *p*-values), one ferroptosis marker gene, SLC40A1 (ferroportin), was significantly upregulated in CMT1A Schwann cells as compared to the age-matched controls (Figure 8). 

**Figure 9:**
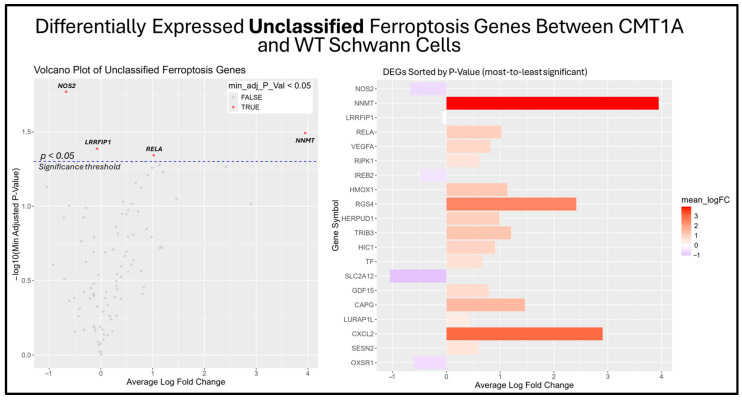
Volcano plot and bar graph showing the top 20 most significant differentially expressed genes (DEGs) between CMT1A and WT Schwann cells in the “Unclassified Ferroptosis” gene set, as listed on FerrDb 2.0 [44]. Transcriptomic data were obtained from Mukherjee-Clavin et al. (2019) [41]. DEGs with adjusted *p*-values and log fold changes (logFCs) were retrieved using GEO2R. The ggplot2 R module was used to graph the results [45]. Significance was determined by an adjusted *p*-value threshold of *p* < 0.05, represented by the dotted blue line in the volcano plot. In both graphs, the X-axis denotes positive (rightward) and negative (leftward) fold changes, while the Y-axis represents -log10(adjusted *p*-value), where higher values indicate greater significance. Gene labels in the volcano plot highlight significant unclassified ferroptosis DEGs in CMT1A Schwann cells.

**Figure 10:**
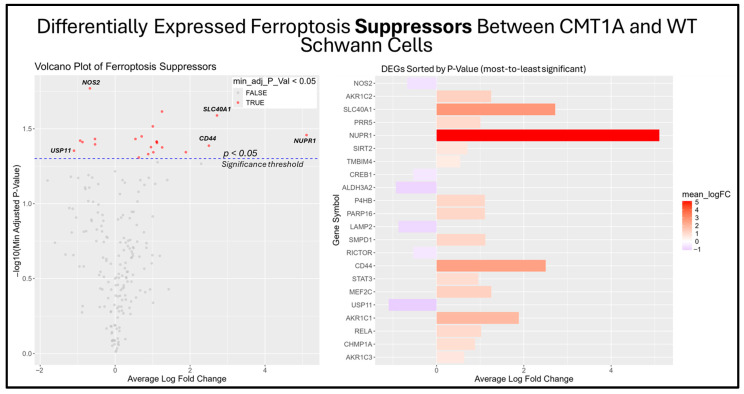
Volcano plots and bar graphs showing all significant differentially expressed genes (DEGs) between CMT1A and WT Schwann cells in the “Ferroptosis Suppressors” gene set, as listed on FerrDb 2.0 [44]. Transcriptomic data were obtained from Mukherjee-Clavin et al. (2019) [41]. DEGs with adjusted *p*-values and log fold changes (logFCs) were retrieved using GEO2R. The ggplot2 R module was used to graph the results [45]. For all analyses, significance was denoted as *p* < 0.05, represented by the dotted blue line in the volcano plot. In both graphs, the X-axis denotes positive (rightward) and negative (leftward) fold changes, while the Y-axis represents -log10(adjusted *p*-value), where higher values indicate greater significance. Labeled genes highlight key ferroptosis suppressors differentially expressed in CMT1A Schwann cells.

Among the unclassified ferroptosis genes, four showed significant differential expression (Figure 9), while all ferroptosis suppressor genes displayed in the bar chart were significantly differentially expressed (Figure 10). These results offer a glimpse into the potential molecular mechanisms behind the elevated activation of the ferroptosis pathway suggested by the GSEA. Among the significantly upregulated unclassified ferroptosis DEGs, nicotinamide N-Methyltransferase (NNMT) and NF-κB p65 (RELA) are of particular interest. NNMT has been described as an inhibitor of GPX4 and SLC7A11, which encodes the light chain portion of system Xc- [51]. The NF-κB pathway, of which RELA participates in, appears to promote ferroptosis through increased lipid peroxidation or the downregulation of system Xc- [52]. In response, many of the significant suppressor DEGs are directly involved in the homeostasis of oxidative stress or iron metabolism. Nitric oxide synthase 2 (NOS2), an enzyme that produces nitric oxide (NO) from L-arginine, is a known ferroptosis suppressor, and shows significant downregulation in CMT1A Schwann cells compared to WT Schwann cells (Figure 9 and Figure 10) [53]. Concurrently, CMT1A Schwann cells show the upregulation of nuclear protein 1 (NUPR1), a well-defined regulator of cellular stress, including oxidative-related stress, and a vital repressor of ferroptosis [54]. Of interest, CMT1A Schwann cells show mixed differential expression of enzymes that regulate the lipid and lipid peroxide metabolism, including the upregulation of the aldo-keto reductase family C1 and C2 (AKR1C1 and AKRC1C2), and the downregulation of aldehyde dehydrogenase 3 family member A2 (ALDH3A2; Figure 9 and Figure 10) [55]. These results reflect the potentially altered iron storage or lipid metabolism in the diseased cell state of CMT1A, which is consistent with our view that compensatory mechanisms against pro-ferroptotic stress are indeed present in CMT1A Schwann cells, as also suggested in our interpretation of Figure 7D.

### 3.5. Pathway Analysis Reveals the Upstream and Downstream Regulators of Ferroptosis in CMT1A Schwann Cells 

**Figure 11:**
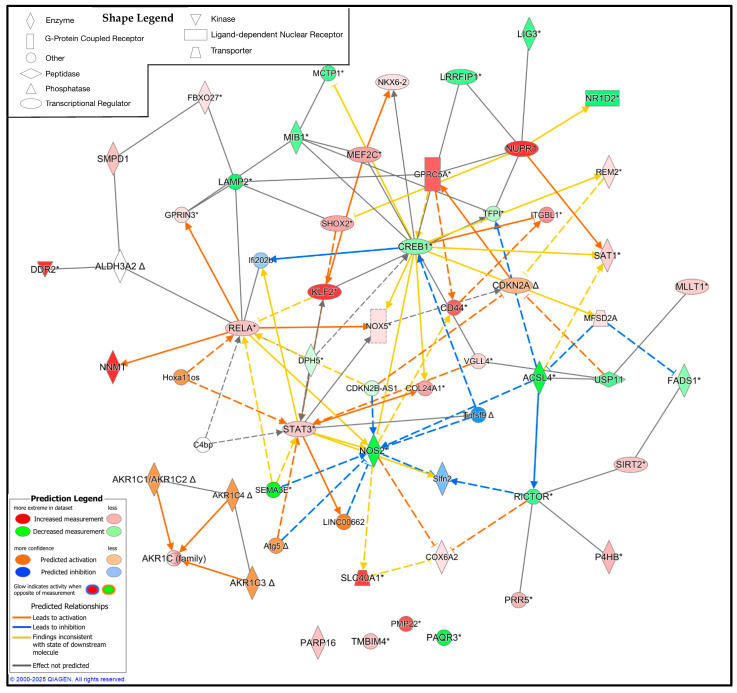
Ingenuity pathway analysis (IPA) of the significant ferroptosis-related differentially expressed genes (DEGs) from the FerrDb V2.0 database between CMT1A and WT Schwann cells, with 25 upstream and downstream regulators included [44]. Significant DEGs were calculated using GEO2R with RNA-Seq data from Mukherjee-Clavin et al. (2019) [41]. Significance of the DEGs was denoted as *p* < 0.05 and log fold changes < 0.5 or > 0.5. The IPA prediction legend is displayed in the bottom left, denoting gene upregulation, downregulation, or predicted activation or inhibition of gene nodes. The shape legend is shown on the top left. Asterisks (*) in the shown pathway denote multiple gene identifiers per node, not statistical significance. Delta symbols (Δ) denote molecules that have undergone a change since the previous IPA release.

While the DEG analysis reveals the altered expression profile of CMT1A Schwann cells in the context of ferroptosis, the specific interplay between these proteins remains unclear. To address this limitation, we utilized QIAGEN’s IPA to identify the potential upstream and downstream regulators of these significant DEGs [46]. We constructed a custom pathway with the significant DEGs from the Mukherjee et al. dataset, shown in Figure 8, Figure 9 and Figure 10, and included 25 upstream or downstream regulators identified by the IPA [41]. Expression data were overlayed on this pathway, revealing the potential central regulators of the identified DEGs (Figure 11). Reflecting the previous results, CMT1A Schwann cells display the altered expression of lipid peroxide-detoxifying enzymes, including ALDH3A2 and the AKR1 family. The IPA additionally revealed NADPH oxidase 5 (NOX5) and acyl-CoA synthetase long-chain family member 4 (ACSL4) as upregulated and downregulated, respectively, further highlighting the altered expression of genes involved in redox homeostasis and lipid metabolism (Figure 11) [56,57]. While ACSL4 is often upregulated with ferroptosis pathway activation, recent data show it is not always required for ferroptosis induction [58,59]. Nuclear factors with significantly altered expression in CMT1A, including Kruppel-like factor 2 (KLF2), cyclic AMP response element-binding protein (CREB1), and signal transducer and activator of transcription 3 (STAT3), potentially show a compensatory state in response to a pro-ferroptotic environment, as all of these transcription factors act as stress regulators and ferroptosis suppressors [60,61,62].

## 4. Discussion

For more than two decades, overexpression of the *Pmp22* gene and its corresponding protein have been established as the primary pathogenic factor driving CMT1A [3]. Despite this understanding, both palliative and curative therapeutic progress has been limited [49]. This is partly due to the elusive function of PMP22; changes in gene dosage ultimately cause cell dysfunction and death, yet the precise cellular mechanisms leading to this outcome remain unclear [3]. Although oxidative stress has long been known as a pathogenic feature of CMT1A, both in human patients and murine models, no studies have demonstrated that the relevant markers, which include elevated lipid peroxides and decreased glutathione, are related to the ferroptosis pathway [7,8,9,10]. 

To the best of our knowledge, this study is the first to demonstrate a connection between ferroptotic stress and CMT1A pathogenesis. Specifically, our results show that human patient-derived CMT1A fibroblasts exhibit heightened susceptibility to ferroptosis induction as compared with age-matched healthy control cells, and show indicators of increased ferroptotic stress at baseline, as measured by a reduction in GPX4 levels and elevated lipid peroxides (Figure 2, Figure 3, Figure 4, Figure 5 and Figure 6). Additionally, data from iPSC-derived CMT1A patient Schwann cells show a significant upregulation of genetic markers, denoting the presence of ferroptosis (Figure 7 and Supplementary Figure S6). Specific ferroptosis genes differentially expressed in CMT1A include regulators of the lipid metabolism (Figure 9 and Figure 10), redox stress (Figure 9 and Figure 10), and iron metabolism, including ferroportin-1, the sole exporter of intracellular iron in the human body (Figure 8), a finding which is consistent with a heightened state of ferroptotic stress [63]. Upstream and downstream analysis of these DEGs supported these results, showing the upregulation of genes related to cellular stress and ferroptotic cell death suppression (Figure 11). 

If ferroptotic stress is indeed a factor in the pathogenesis of CMT1A, it is helpful to consider the role of ferroptosis in the CNS and its disorders, as this has been studied to a greater extent than the PNS. Evidence for ferroptosis activity, including increased LPO, increased iron stores, decreased GPX4, and decreased glutathione, is present in several CNS diseases, such as Alzheimer’s disease and Parkinson’s disease [13,19,29]. This ferroptosis activity may be exacerbated by low-level, sterile inflammation caused by damage-associated molecular patterns (DAMPs) released by ferroptotic cells [14,15,16,21,22,23,24,31]. For instance, inflammatory molecules, such as interleukin-6 (IL-6), γ-interferon, and tumor necrosis factor alpha (TNF-α), all have an adverse effect on GPX4, and IL-6 itself shows evidence of inducing lipid peroxidation and promoting iron imbalance in studies on chondrocytes [64,65,66]. A pro-inflammatory environment could cause more neurons to undergo ferroptotic stress, which would generate additional sources of lipid peroxides and create a feed-forward mechanism of neurodegeneration that manifests as symptomatic disease [13,67,68,69].

**Figure 12:**
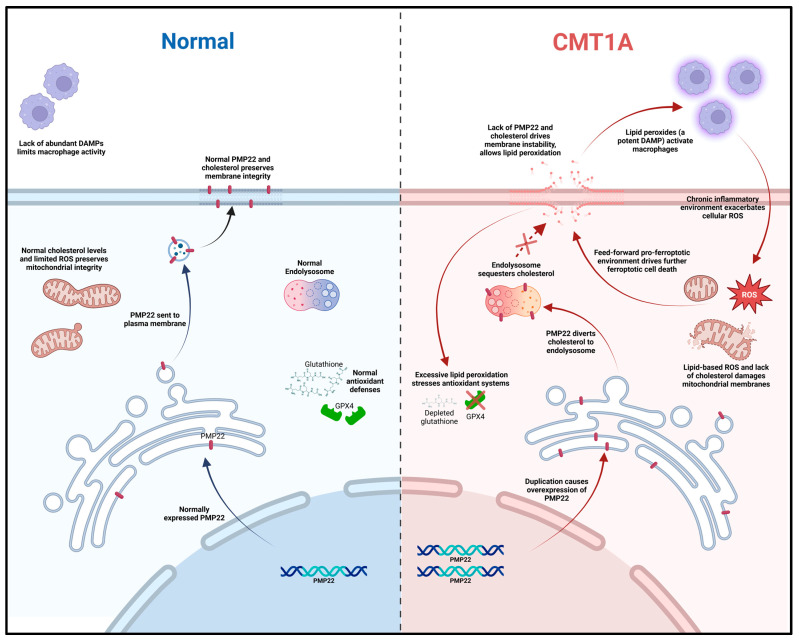
Mechanistic diagram illustrating our working hypothesis of ferroptosis susceptibility in CMT1A. A duplication in the *PMP22* gene increases the expression of the PMP22 protein, causing the aberrant sub-cellular trafficking of both PMP22 itself and cholesterol to the endo-lysosomal system. Loss of mitochondrial membrane integrity from aberrant cholesterol content causes leakage in the electron transport chain, decreasing the efficiency and driving lipid-based ROS production; concurrently, plasma membrane instability allows for the release of phospholipids and lipid-based ROS into the extracellular space, activating native macrophages and fostering chronic inflammation in neural tissue. Chronic, low-level sterile inflammation with aging causes the decompensation of Schwann cells, leading to symptoms of late-stage CMT1A.

Based on current literature and our own data, we propose a working model for ferroptosis involvement in CMT1A. In this model, overexpressed PMP22, which normally traffics to the plasma membrane, is diverted to the lysosome, which diverts cholesterol in the process [4]. This causes lysosomal cholesterol sequestration, which disrupts the maintenance of antioxidant and mitochondrial homeostasis, potentially through membrane dysfunction which causes excessive lipid peroxidation (Figure 12) [4,70,71]. Alternatively, the underexpression of PMP22, characteristic of HNPP, a disease with similar clinical presentation to CMT1A, leads to cholesterol sequestration in the Golgi apparatus, as PMP22 appears necessary for anterograde cholesterol transport [3,4]. These perturbations lead to compromised antioxidant defenses, resulting in GPX4 downregulation and lipid peroxide accumulation (Figure 4, Figure 5 and Figure 6); these lipid peroxides may disrupt membrane integrity causing death in susceptible cells. In the early stages of the disease, most cells may compensate for these defects in antioxidant systems (Figure 1 and Figure 7, Figure 8, Figure 9, Figure 10 and Figure 11) [72]. However, age-related damage would likely exacerbate these vulnerabilities, progressively lowering the antioxidant defense baseline and increasing the likelihood of ferroptotic cell death (Figure 1, Figure 2, Figure 3, Figure 4, Figure 5 and Figure 6) [72]. Our transcriptomic analysis showed a significant upregulation of protective ferroptosis components (Figure 7 and Figure 10), which may be explained by the use of iPSC-derived Schwann cells, as this process reverts cells to an embryonic form which could undo age-related damage to a certain degree, allowing for more robust anti-ferroptosis protection [73,74]. This is in contrast with our in vitro work with somatic cells from 51- and 40-year-old subjects, which show evidence of compromised antioxidant defenses at baseline and after exposure to ferroptotic stress through RSL3 (Figure 2, Figure 3, Figure 4, Figure 5 and Figure 6). With progressive age, we believe that cellular decompensation would manifest as late-stage symptoms of CMT1A, including the loss of motor function and quadriplegia [2].

Given the evidence of ferroptosis involvement in CMT1A, targeted anti-ferroptotic therapies could stabilize the age-related decline in antioxidant defenses, possibly extending motor function in affected patients [2,3]. The anti-ferroptotic drugs J147 and CMS121 show promise for treating Alzheimer’s disease, and might be re-purposed for PNS disorders, including CMT1A [25,26,27,28,29,30,31]. Curcumin, a less potent precursor to J147, has already been shown to ameliorate the clinical phenotype of CMT1A in a murine model, offering early evidence for ferroptosis-targeted drug therapies for CMT1A [8].

As this study is limited in scope, future work should involve a more comprehensive analysis of ferroptosis susceptibility in CMT1A cells. This includes evaluating key components of the ferroptosis pathway at different stages of disease progression, such as system Xc-, GPX4–protein interactions, intracellular iron regulation, and glutathione metabolism. Investigating these components in vivo would also yield valuable data on the role of ferroptosis in CMT1A pathogenesis.

## 5. Conclusions

This study provides the first documented evidence linking ferroptosis to CMT1A pathogenesis, and the first to associate ferroptotic stress with a hereditary condition of the peripheral nervous system. Our findings demonstrate that cultured CMT1A fibroblasts exhibit compromised antioxidant defenses, while transcriptomic data from CMT1A Schwann cells suggest a compensatory response to chronic ferroptotic stress. These results identify ferroptosis as a novel pathogenic mechanism in CMT1A, which may serve as a potential target for anti-ferroptotic therapies for this disease and related peripheral neuropathies.

## Data Availability

All data are contained within this article and Supplementary Materials.

## Supplementary Materials

The following supporting information can be downloaded at: https://www.mdpi.com/article/10.3390/antiox14030331/s1, Figure S1: MTT assay of 40-year-old cell lines with dose–response; Figure S2: MTT assay of 51-year-old cell lines with 24-h RSL3 treatment; Figure S3: Cell counting verification with 40-year-old cell lines; Figure S4: BODIPY-C11 lipid peroxidation staining with 40-year-old cell lines; Figure S5: Quantification of BODIPY-C11 lipid peroxidation staining with 40-year-old cell lines; Figure S6: Ferroptosis enrichment plots of an independent dataset from Li et al. [41]; Figure S7: Full Western blot images of GPX4 from 51-year-old cell lines.

## Abbreviations

The following abbreviations are used in this manuscript:

CMT	Charcot–Marie–Tooth disease
CMT1A	CMT type 1A
PMP22	peripheral myelin protein 22
HNPP	hereditary neuropathy with liability to pressure palsies
RSL3	RAS-selective lethal 3
ROS	reactive oxygen species
LPOs	lipid peroxides
GPX4	glutathione peroxidase 4
NNMT	nicotinamide N-methyltransferase
NF-κB	nuclear factor kappa-light-chain-enhancer of activated B cells
RELA	v-rel reticuloendotheliosis viral oncogene homolog A
NOS2	NF-κB p65 subunit nitric oxide synthase 2
NOX5	NADPH oxidase 5
ACSL4	Acyl-CoA synthetase long-chain family member 4
AKR1C1/2	aldo-keto reductase family C1 and C2
ALDH3A2	aldehyde dehydrogenase 3 family member A2
IL6	interleukin-6
TNF-α	tumor necrosis factor alpha
DAMPs	damage-associated molecular patterns
GSEA	gene set enrichment analysis
IPA	ingenuity pathway analysis
iPSC	induced pluripotent stem cell
