# Supplementary material for: Ferroptosis and Charcot–Marie–Tooth Disease 1A: Emerging Evidence for a Pathogenic Association
Source: Antioxidants (Basel). 2025 Mar 11;14(3):331. doi: 10.3390/antiox14030331 (PMC11939244; doi:10.3390/antiox14030331)
Supplement: Supplementary file 1 [file antioxidants-14-00331-s001.zip › antioxidants-3499652-supplementary-final.pdf]

Supplementary Figures

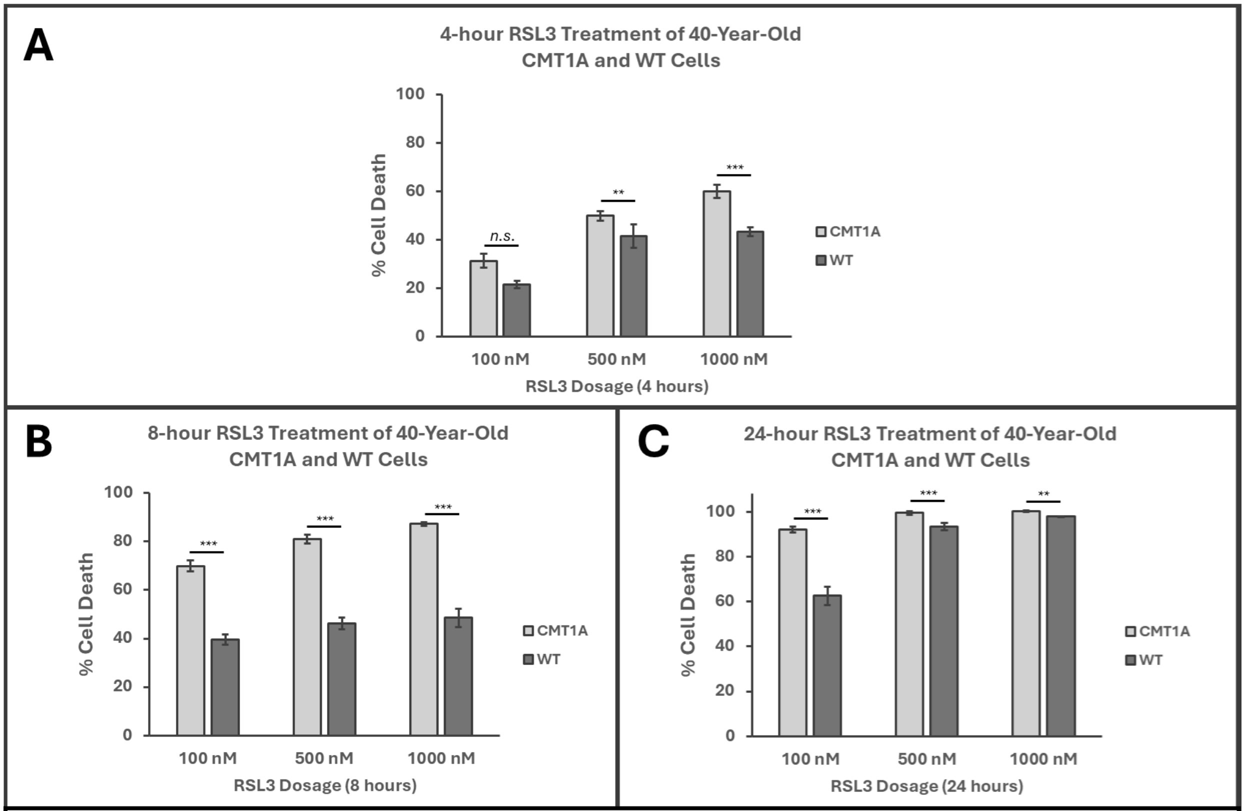

Supplemental Figure S1. MTT cell toxicity assay of patient-derived, age-matched, 40-year-old CMT1A and control (wild-type; WT) primary fibroblasts exposed to varying doses of Ras-selective lethal 3 (RSL3), a selective inhibitor of glutathione peroxidase 4 (GPX4) and pro-ferroptotic chemical. Cells were seeded at a density of 2000 per well in 96-well plates for 24-48 hours. Cells were treated with RSL3 at doses of 100, 500, and 1000 nM. After 4 (**A**), 8 (**B**), or 24 hours (**C**), the pertinent treatments were removed. Cells were then incubated with MTT reagent for three hours. After solubilizing cells with 5% sodium-dodecyl-sulfate (SDS) in dimethyl sulfoxide (DMSO), well absorbance values were read with a plate reader at 590 nm. Error bars display standard error of the mean. Data shown were derived from a single independent experiment with octuplicates per treatment group. Error bars show standard error of the mean. An unpaired Student's *t*-test was used to calculate statistical differences (** $p < 0.01$; *** $p < 0.001$).

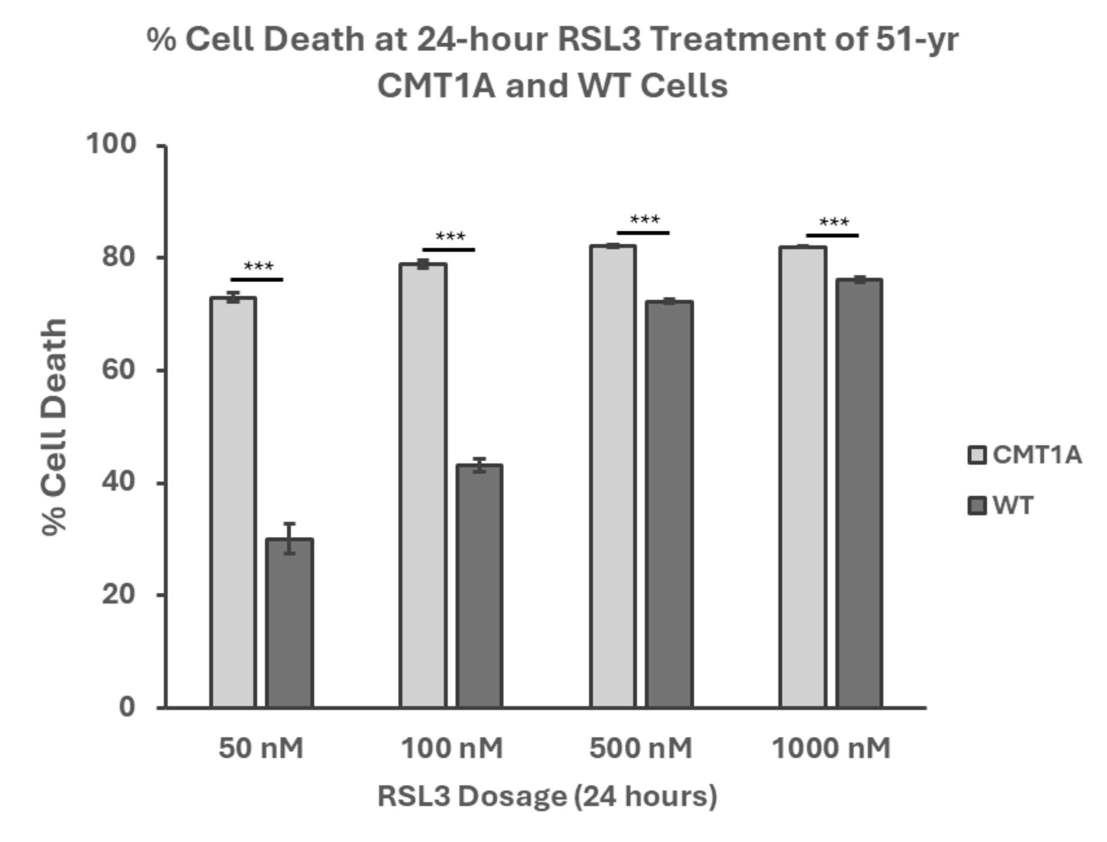

Supplemental Figure S2. MTT cell toxicity assay of patient-derived, age-matched, 51-year-old CMT1A and control (wild-type; WT) primary fibroblasts exposed to varying doses of Ras-selective lethal 3 (RSL3), a selective inhibitor of glutathione peroxidase 4 (GPX4) and pro-ferroptotic agent, for 24 hours. Cells were seeded at a density of 2000 per well in 96-well plates for 24-48 hours. Cells were treated with RSL3 at doses of 50, 100, 500, and 1000 nM. After 24 hours, the pertinent treatments were removed; cells were incubated with MTT reagent for three hours. After solubilizing cells with 5% sodium-dodecyl-sulfate (SDS) in dimethyl sulfoxide (DMSO), well absorbance values were read with a plate reader at 590 nm. Error bars display standard error of the mean. Error bars show standard error of the mean. Data are from a single independent experiment with octuplicates per group. An unpaired Student's *t*-test was used to calculate statistical differences. *** $p < 0.001$.

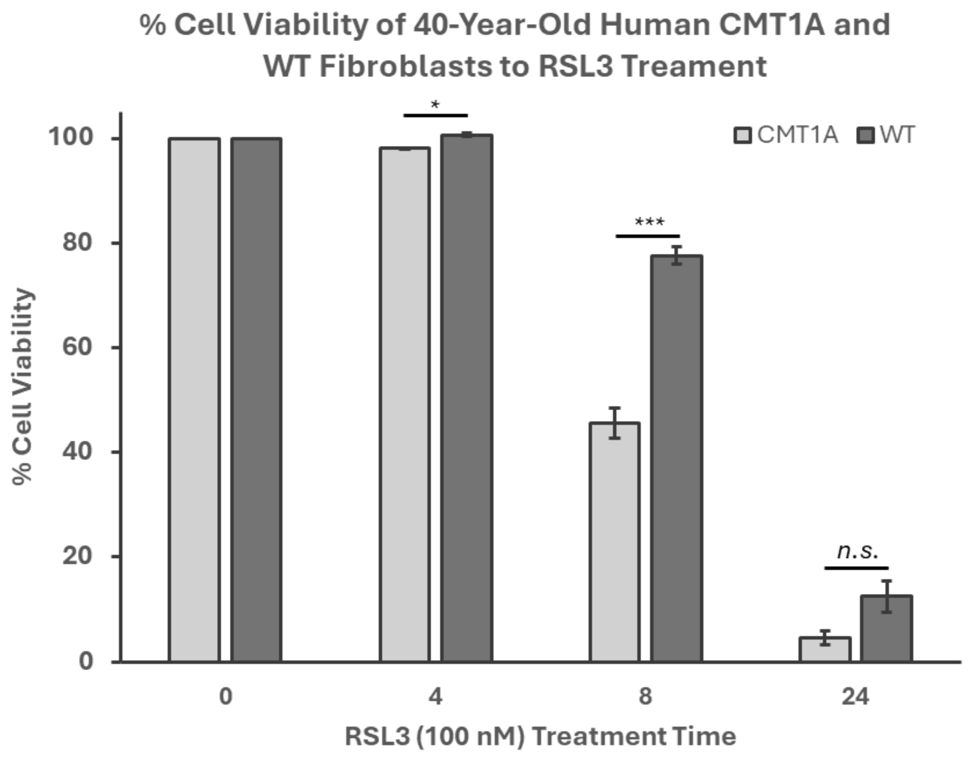

Supplemental Figure S3. Percent cell viability of Charcot-Marie-Tooth disease type 1A (CMT1A) and wild-type (WT; control) primary fibroblasts derived from 40-year-old human patient donors at 0-24 hours of treatment with Ras-selective lethal 3 (RSL3), a pro-ferroptotic inducer and an inhibitor of glutathione peroxidase 4 (GPX4). Cells were seeded at 40,000 cells/well for 24-72 hours. Cell medium was then aspirated and replaced with 100 nM of RSL3. After 4, 8, and 24 hours, cells were imaged using an Invitrogen EVOS Digital Color Fluorescence Microscope at 4x magnification. Cell counts were performed manually for all groups, and statistical analysis was performed in Microsoft Excel. Data are from a single independent experiment in triplicate. Error bars signify standard error of the mean. Student's *t*-test was used to calculate statistical significance. * $p < 0.05$, *** $p < 0.001$, *n.s.* = no significance.

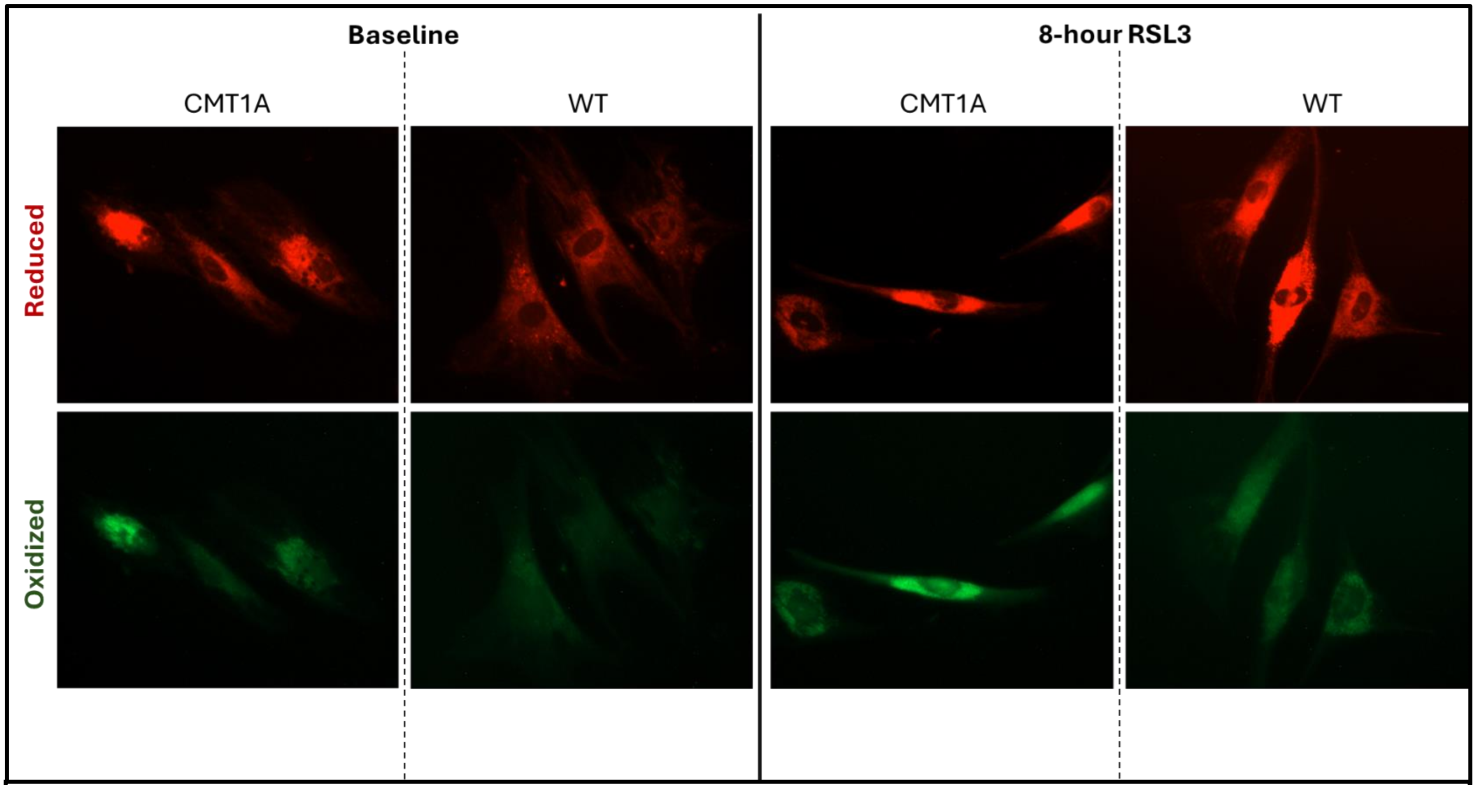

Supplemental Figure S4. Fluorescent microscopy images (10× magnification) of 40-year-old Charcot-Marie-Tooth disease type 1A (CMT1A) and wild-type (WT; control) human-derived fibroblasts stained with BODIPY™ 581/591 C11 undecanoic acid, a lipid peroxidation sensor. BODIPY C11 acts as a lipid peroxidation sensor, as oxidation of this lipid's polyunsaturated butadienyl moiety shifts the fluorescence emission peak from red (590 nm) to green (510 nm). After CMT1A or WT cells were treated with medium alone (vehicle control) or 100 nM RSL3 for 8 hours, BODIPY C11 staining was performed at 37°C in a cell incubator for 30 minutes before fixation with 4% paraformaldehyde.

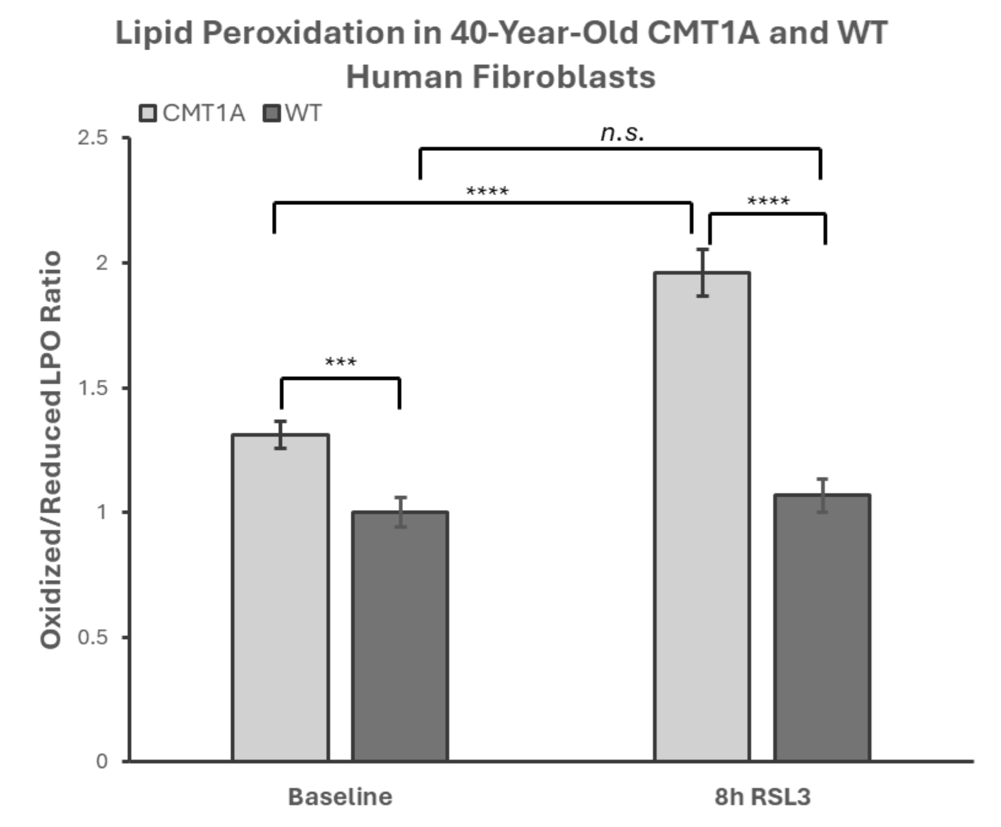

Supplemental Figure S5. Quantification of green-to-red emission (oxidized-to-reduced) ratio from BODIPY™ 581/591 C11 undecanoic acid staining of 40-year-old Charcot-Marie-Tooth disease type 1A (CMT1A) and wild-type (WT; control) human-derived fibroblasts, before and after ferroptosis induction with RSL3. Fibroblasts were treated with either growth medium (vehicle control) or 100 nM RSL3 for 8 hours, prior to a 30-minute incubation at 37°C with 1 µM BODIPY™ 581/591 C11 and immediate fixation with 4% paraformaldehyde. Green and red emission images were captured with a digital fluorescent microscope and quantified in ImageJ software. Statistical analysis was performed in Microsoft Excel. Error bars denote standard error of the mean. *** $p < 0.001$, **** $p < 0.0001$.

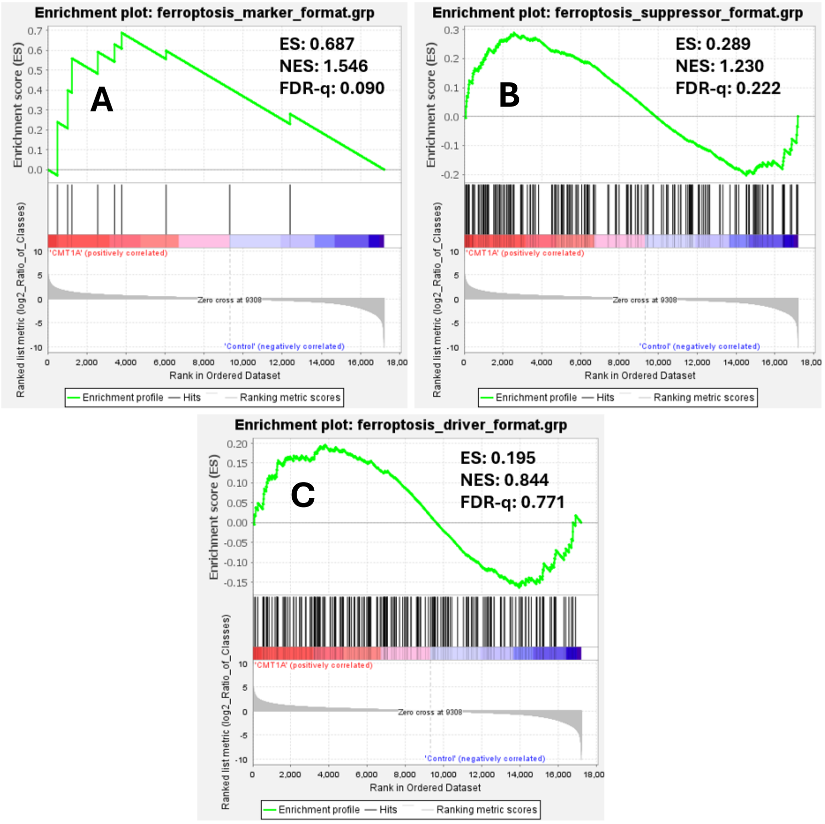

Supplemental Figure S6. Enrichment plots of ferroptosis markers (A), suppressors (B), and drivers (C) in CMT1A iPSC-derived Schwann cells compared with wild-type (WT) controls [42]. Based on the GSEA developer recommendation of an FDR-q <0.25, ferroptosis markers and suppressors show significant upregulation and enrichment compared with controls, but not ferroptosis drivers or unclassified genes. Ferroptosis gene sets were retrieved from FerrDb V2.0 [44].

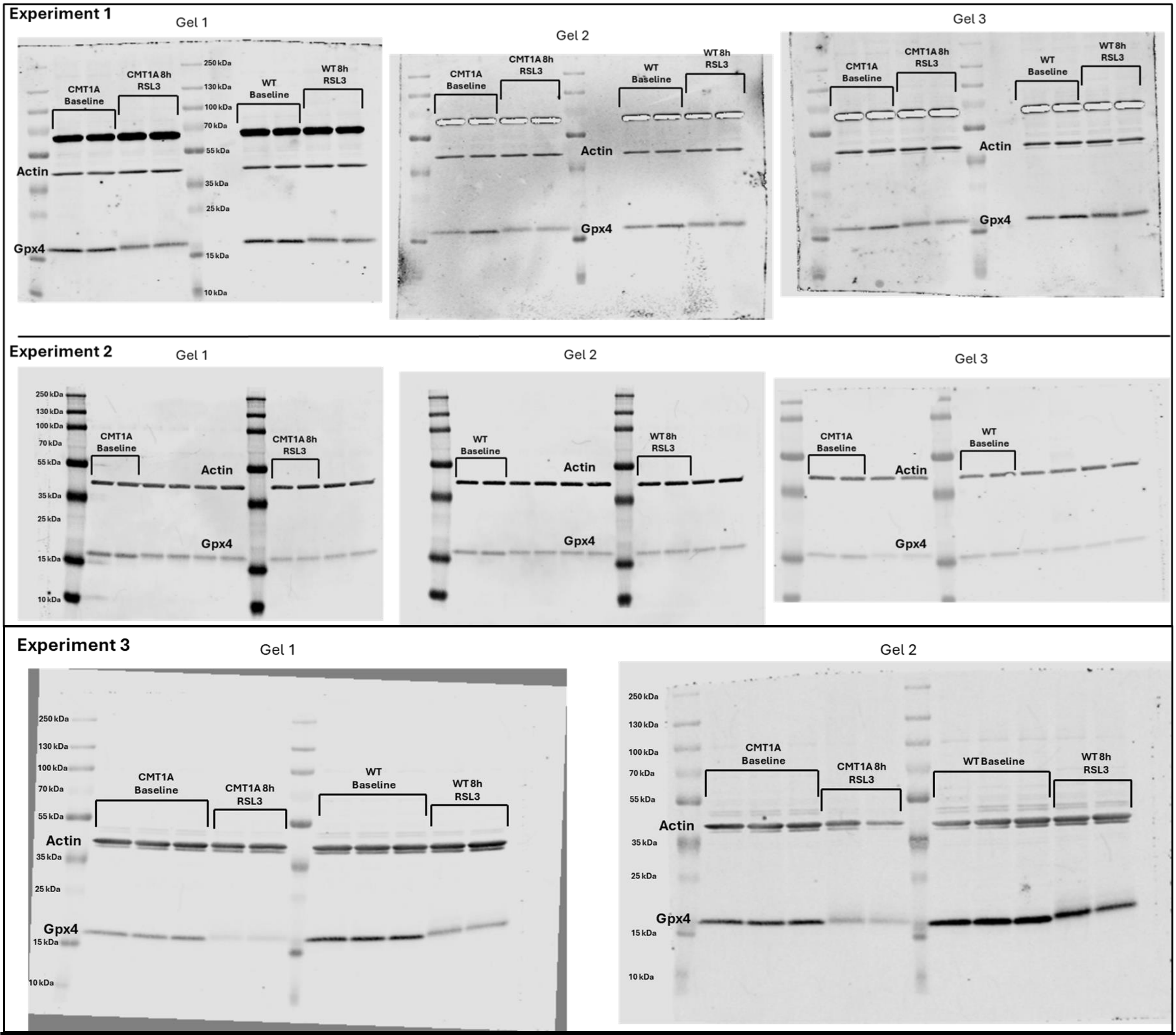

Supplemental Figure S7. Western blot images human-derived fibroblasts from 51-year-old Charcot-Marie-Tooth disease type 1A (CMT1A) patients and wild-type (WT) control patients. Representative Western blot images of GPX4 and actin (loading control) showing either medium alone (vehicle control) or 100 nM RSL3 for 8 hours. Cell lysates were separated by SDS-PAGE and transferred onto a nitrocellulose membrane, followed by incubation with primary antibodies (1:1000, 24 hours, 4°C) and secondary antibodies (1:10,000, 1 hour, room temperature). Images were captured in Image Studio 5.5 using an Odyssey CLx Western blot machine.
